# The Antioxidative Function of Alpha-Ketoglutarate and Its Applications

**DOI:** 10.1155/2018/3408467

**Published:** 2018-03-21

**Authors:** Shaojuan Liu, Liuqin He, Kang Yao

**Affiliations:** ^1^Key Laboratory of Agro-Ecological Processes in Subtropical Region, Institute of Subtropical Agriculture, Chinese Academy of Sciences, National Engineering Laboratory for Pollution Control and Waste Utilization in Livestock and Poultry Production, Hunan Provincial Engineering Research Center for Healthy Livestock and Poultry Production, Changsha, Hunan 410125, China; ^2^University of Chinese Academy of Sciences, Beijing 100049, China

## Abstract

Alpha-ketoglutarate (AKG) is a crucial intermediate of the Krebs cycle and plays a critical role in multiple metabolic processes in animals and humans. Of note, AKG contributes to the oxidation of nutrients (i.e., amino acids, glucose, fatty acids) and then provides energy for cell processes. As a precursor of glutamate and glutamine, AKG acts as an antioxidant agent as it directly reacts with hydrogen peroxide with formation of succinate, water, and carbon dioxide; meanwhile, it discharges plenty of ATP by oxidative decarboxylation. Recent studies also show that AKG has alleviative effect on oxidative stress as a source of energy and an antioxidant in mammalian cells. In this review, we highlight recent advances in the antioxidative function of AKG and its applications in animals and humans.

## 1. Introduction

Reactive oxygen species (ROS) are oxygen-containing chemical species including superoxide anion, hydrogen peroxide (H_2_O_2_), and hydroxyl radicals, and most of which are produced by mitochondria and nicotinamide adenine dinucleotide phosphate (NADPH) oxidases [1]. Of note, excess of ROS could lead to oxidative stress in cells. Oxidative stress is associated with the disorder of proteins, lipid oxidation, and nucleic acid breaks, which may further impair cellular physiological functions. Numerous studies suggested that oxidative stress may result in some pathogenic diseases, such as cancer [2], neurological disorders [3], age-related diseases [4], atherosclerosis [5], inflammation [6], and cardiovascular diseases [7]. Mammals have evolved a series of antioxidant defenses to protect vital biomolecules from oxidative damage. On the one hand, antioxidant agents, such as antioxidant enzymes like superoxide dismutase (SOD), catalase (CAT), and glutathione peroxidase (GSH-Px), or nonenzymatic agents, such as glutathione (GSH), vitamin C, and vitamin E, can clean off most of ROS [8]. On the other hand, the excess ROS can also activate many signaling pathways such as mitogen-activated protein kinase (MAPKs), NF-erythroid 2-related factor/antioxidant response element (Nrf2/ARE), and peroxisome proliferator-activated receptor *γ* (PPAR*γ*), which play a vital role in cellular redox homeostasis and contribute to antioxidative defense [9].

Glutamate, as a precursor of GSH, exerts alleviative effects on oxidative stress in medicine and surgery [10]. AKG, as a precursor of glutamine, is cheaper and more stable than glutamine and acts as an antioxidant instead of glutamine in many cellular processes. Many reports demonstrated that AKG can be converted into glutamine by glutamate dehydrogenase (GDH) and glutamine synthetase (GS), which is a sign of antioxidative function. It is evident that AKG could improve antioxidative capacity by promoting glutamine content and antioxidative systems [11, 12]. Additionally, Chen et al. showed that AKG could significantly improve SOD activity but reduce malondialdehyde (MDA) level, suggesting an improvement of intestinal antioxidative capacity [13]. Recently, more and more studies indicated that AKG could improve antioxidative function against oxidative imbalance in cells, which further contributed to the prevention and treatment of various diseases induced by oxidative stress. Therefore, in this review, we aim to summarize the recent advances of the antioxidative function of AKG and its applications.

## 2. Biochemical Characteristics of AKG

**Figure 1:**
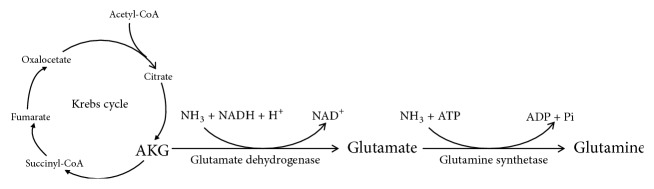
The conversion of AKG into glutamate and glutamine.

**Figure 2:**
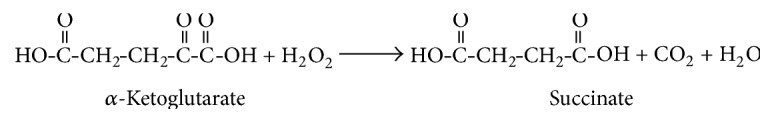
Nonenzymatic oxidative decarboxylation of AKG in hydrogen peroxide decomposition.

AKG is a weak acid containing two carboxyl groups and a ketone group which is also called 2-ketoglutaric acid or 2-oxoglutaric acid. AKG possesses many physiological functions. On the one hand, AKG could react with ammonia and then be converted into glutamate; subsequently, the glutamate further reacts with ammonia and generates glutamine (Figure 1). On the other hand, AKG reacts with H_2_O_2_ as a result of the conversion of succinate, carbon dioxide (CO_2_), and water (H_2_O), eventually achieving elimination of H_2_O_2_ (Figure 2) [14]. Additionally, AKG could produce plenty of ATP in the TCA cycle and provide energy for intestinal cell processes. Furthermore, AKG performs positive effects on oxidative stress damage in intestinal mucosal cells and contributes to cell redox homeostasis [15]. It has been reported that enteral AKG was oxidized and used by intestinal mucosa, thereby, as an energy donor and antioxidant agent via the TCA cycle. Apart from the above, AKG also exerts antioxidative defense by enzymatic systems and nonenzymatic oxidative decarboxylation.

## 3. Antioxidative Function of AKG

### 3.1. Antioxidants Activities

The balance between oxidants and antioxidants plays an important role in physiological functions in cells and biomolecules. Antioxidant system comprises enzymatic and nonenzymatic agents. Antioxidative enzymes include SOD, CAT, GSH-Px, and nonenzymatic agents include GSH, vitamin C, vitamin E [10]. AKG is an antioxidant substance which exhibits a vital role in scavenging ROS in organism [16]. Growing studies suggest that AKG serves as a natural antidote of scavenging ammonia by exerting its antioxidative capacity. It has been reported that AKG inhalation showed a protective role in ammonia-induced lung damage in rats [17]. The mechanism may be caused by reducing the levels of lactate dehydrogenase (LDH) and MDA and improving the activities of SOD and CAT and GSH level. Lipid peroxidation is susceptible to ammonia or trauma like burns and eventually produces MDA resulting in membrane injury and even cell apoptosis, while antioxidants such as SOD and GSH-Px are beneficial to prevent the lipid peroxidation and injury [18]. AKG could prevent the lipid peroxidation by increasing SOD, GSH-Px, and CAT activities to facilitate fat metabolism, and then alleviate ethanol-induced hepatotoxicity and hyperammonemia induced by ammonium acetate in rats [19, 20]. Similarly, AKG also performs chemopreventive role in hepatocarcinogenesis induced by N-nitrosodiethylamine (NDEA) in rats by modulating the levels of antioxidants and lipid peroxide to access normal levels [21]. Furthermore, AKG shows high resistance to ammonia-N stress in hybrid sturgeons as it enhances antioxidant enzymes activity and HSP 70 and HSP 90 gene expression [22]. Besides, cyanide-induced oxidative stress could lead to neurotoxicity, the lipid peroxidation, and dysfunction of membrane especially in brain and kidney of animals like rats [23]. And cyanide is evident to inhibit antioxidative defense such as reducing SOD activity and GSH level [24]. Interestingly, AKG is considered as a natural antagonist of cyanide poisoning because of its chemical structure that is able to bind with cyanide to produce cyanohydrin and further prevent cyanide poisoning or cyanide lethality [25, 26]. In rat in vitro and vivo models, AKG reduces GSH depletion and DNA damage induced by cyanide [27]. Furthermore, studies demonstrate that AKG alone could prevent brain and liver from cyanide-induced oxidative damage by increasing GSH, SOD, and GSH-Px levels and reducing MDA level in rats, especially when combined with sodium thiosulfate [28, 29]. Additionally, a recent study indicates that AKG could enhance freeze-thaw tolerance and prevent cell death induced by carbohydrate stress in yeast, and the protective pathway may be involved in the enhanced antioxidant defense [30].

### 3.2. Nonenzymatic Oxidative Decarboxylation in H_2_O_2_ Decomposition

In regard of antioxidative defense, some studies show that AKG exerts its function by other redox regulatory mechanisms rather than antioxidant activities. A number of studies demonstrate that AKG acts as a source of energy and antioxidant agent on improving physiological metabolism and scavenging ROS to alleviate oxidative stress via nonenzymatic oxidative decarboxylation in H_2_O_2_ decomposition. Hydrogen peroxide, one of ROS, is a weak oxidant and cytotoxic and easily causes oxidative stress injury in cells such as cell membrane damage and DNA alterations [31]. Indeed, pyruvate and *α*-ketoacids exhibit protective effects on H_2_O_2_-induced toxicity in vivo and vitro and can cross the blood-brain barrier and scavenge H_2_O_2_, which provide a novel therapeutic mode against H_2_O_2_-induced brain pathologies. The mechanism may be due to the nonenzymatic oxidative decarboxylation in which ketone group in *α*-carbon atom is combined with H_2_O_2_ to form corresponding carboxylic acid, CO_2_, and H_2_O. AKG serves as a key intermediate in the TCA cycle and participates in nonenzymatic oxidative decarboxylation in the H_2_O_2_ decomposition. It has been demonstrated that AKG significantly elevated antioxidative capacity by decreasing the level of H_2_O_2_ in the liver and intestinal mucosa of ducks [32]. Also, AKG performs a protective role in intestinal cells damage induced by H_2_O_2_ through mitochondria pathway [33]. Similarly, the protective action of AKG is noticed in alleviating toxic effects of H_2_O_2_ in* Drosophila melanogaster*, other animals, and humans, which provides a strong evidence for the H_2_O_2_-scavenging ability of AKG [34]. Thus, AKG can be used as a potent scavenger in nonenzymatic oxidative decarboxylation in H_2_O_2_ decomposition.

## 4. The Applications of AKG in Animals and Humans

AKG has been widely used in animals and humans as a feed additive and medicine. In animal industry, AKG could effectively improve growth performance, nitrogen utilization, immunity, bone development, intestinal mucosal injury, and oxidative system [35–39]. In humans, AKG is extensively used in trauma, aged diseases, postoperative recovery, and other nutritional diseases [40]. In terms of antioxidative function, AKG exhibits a crucial role in multiple diseases involved in aging, cancer, cardiovascular diseases, and neurological diseases. It has been reported that AKG developed its antioxidant capacity to fight against ethanol toxicity and enhance cold tolerance in the model of* Drosophila*, which provided an effective therapy against ethanol and alcohol poisoning in animals and humans [41, 42]. Similar protective effect is noticed in lipopolysaccharide-induced liver injury in which AKG provides a new intervention to alleviate liver damage in young pigs [43]. AKG also maintains redox state stabilization for antioxidant defense. Indeed, AKG oxidation plays a beneficial role in maintaining the levels of reductive carboxylation to handle mitochondrial defects in cancer cells [44]. Besides, oral administration of AKG improves blood vessel elasticity by exerting its antioxidant in aging organisms [45]. Additionally, AKG could facilitate the rate of GSH synthesis in human erythrocytes [46]. AKG has been identified to effectively decrease the incidence of cataracts induced by sodium selenite in rat and acted as a scavenger of ROS [47]. Moreover, AKG functions as a neuroprotective agent in ischemic pathology of hippocampus [48]. Furthermore, a novel study demonstrates that AKG could regulate organismal lifespan and prevent age-related diseases by regulating cellular energy metabolism [49]. Interestingly, apart from antioxidative function, AKG is characterized by prooxidative property which can generate active complexes with iron in rat brain homogenates [50, 51]. Under mild oxidative stress, it results in activating antioxidant system of AKG, thus displaying its protective effects such as strengthening resistance of the yeast cells to oxidative stress [52].

## 5. Summary and Perspective

**Figure 3:**
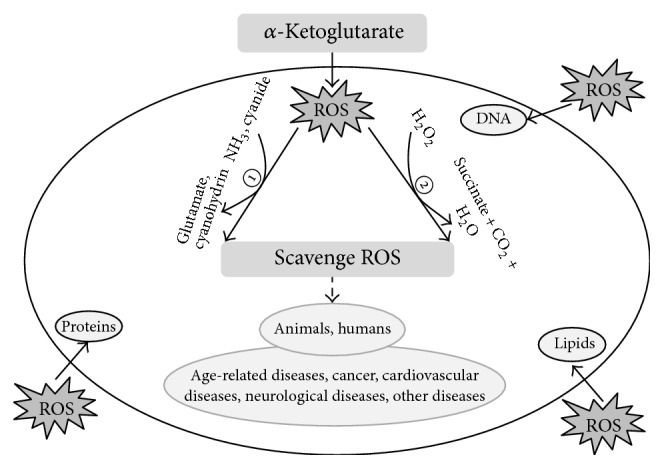
The antioxidative function of AKG and its applications. ①: antioxidative enzymes activites; ②: nonenzymatic oxidative decarboxylation in hydrogen peroxide decomposition.

AKG serves as a pivotal intermediate and is widely applied in animals and humans. Particularly, AKG primarily exerts its antioxidative function by the following: (1) enhancing antioxidative enzymes activities and nonenzymatic agent levels against oxidative stress and lipid peroxidation, especially in intervention of ammonia and cyanide poisoning; (2) participating in nonenzymatic oxidative decarboxylation in H_2_O_2_ decomposition to scavenge ROS and protect organism from various ROS-induced diseases. And AKG provides a promising therapeutic intervention for clinical diseases in animals and humans (Figure 3). Besides the above antioxidative pathways, Nrf2/ARE is an important regulator of antioxidative process that aids to keep redox homeostasis, and it has been proved to perform a vital role in various diseases (i.e., liver injury, traumatic brain injury, and inflammation) induced by oxidative stress [53]. Of particular interest, glutamine has been verified to improve the gene expression of Nrf2 by activating Nrf2/ARE signaling pathway to suppress ROS generation, elevate GSH levels, and prevent apoptosis in intestine [54, 55]. However, as a precursor of glutamine, whether AKG could directly activate Nrf2/ARE signaling pathway to alleviate oxidative stress or not, relevant research about that is not reported and further study is needed.

## Abbreviations

AKG: Alpha-ketoglutarate
ROS: Reactive oxygen species
H_2_O_2_: Hydrogen peroxide
NADPH: Nicotinamide adenine dinucleotide phosphate
SOD: Superoxide dismutase
CAT: Catalase
GSH-Px: Glutathione peroxidase
GSH: Glutathione
MAPKs: Mitogen-activated protein kinase
Nrf2/ARE: NF-Erythroid 2-related factor/antioxidant response element
PPAR*γ*: Peroxisome proliferator-activated receptor *γ*
GDH: Glutamate dehydrogenase
GS: Glutamine synthetase
MDA: Malondialdehyde
CO_2_: Carbon dioxide
H_2_O: Water
LDH: Lactate dehydrogenase
NDEA: N-Nitrosodiethylamine.

## References

[1] Scherz-Shouval R., Elazar Z. (2011). Regulation of autophagy by ROS: physiology and pathology. *Trends in Biochemical Sciences*.

[2] Dharmaraja A. T. (2017). Role of reactive oxygen species (ROS) in therapeutics and drug resistance in cancer and bacteria. *Journal of Medicinal Chemistry*.

[3] Cheignon C., Tomas M., Bonnefont-Rousselot D., Faller P., Hureau C., Collin F. (2018). Oxidative stress and the amyloid beta peptide in Alzheimer’s disease. *Redox Biology*.

[4] Abdollahi M., Moridani M. Y., Aruoma O. I., Mostafalou S. (2014). Oxidative stress in aging. *Oxidative Medicine and Cellular Longevity*.

[5] Essick E. E., Sam F. (2010). Oxidative stress and autophagy in cardiac disease, neurological disorders, aging and cancer. *Oxidative Medicine and Cellular Longevity*.

[6] Bryk D., Olejarz W., Zapolska-Downar D. (2017). The role of oxidative stress and NADPH oxidase in the pathogenesis of atherosclerosis. *Postepy Higieny I Medycyny Doswiadczalnej*.

[7] Chen K., Keaney J. F. (2012). Evolving concepts of oxidative stress and reactive oxygen species in cardiovascular disease. *Current Atherosclerosis Reports*.

[8] Reuter S., Gupta S. C., Chaturvedi M. M., Aggarwal B. B. (2010). Oxidative stress, inflammation, and cancer: how are they linked?. *Free Radical Biology & Medicine*.

[9] Jha N., Ryu J. J., Choi E. H., Kaushik N. K. (2017). Generation and Role of Reactive Oxygen and Nitrogen Species Induced by Plasma, Lasers, Chemical Agents, and Other Systems in Dentistry. *Oxidative Medicine Cellular Longevity*.

[10] Schemitt E. G., Colares J. R., Hartmann R. M. (2016). Effect of glutamine on oxidative stress and inflammation in a rat model of fulminant hepatic failure. *Nutrición Hospitalaria*.

[11] Wang L., Qiyou X. U., Chen D., Zheng R. (2016). Effects of Dietary *α*-Ketoglutarate Supplementation on Liver Glutamine Content,Antioxidant Capacity and the Expressions of Growth Hormone and Insulin-Like Growth Factor I Genes of Juvenile Hybrid Sturgeon Fed Different Protein Source Diets. *Chinese Journal of Animal Nutrition*.

[12] Song F., Wang L., Qiyou X. U., University N. A. (2016). Effects of Glutamine and Its Precursors on Tissue Antioxidant Capacity and Serum Biochemical Indices of Songpu Mirror Carp. *Chinese Journal of Animal Nutrition*.

[13] Chen D., Wang L. S., Qi-You X. U. (2015). Effects of *α*-ketoglutarate supplementation on morphology, digestive enzyme activity and antioxidant capacity in intestine of hybrid sturgeon. *Journal of Dalian Ocean University*.

[14] Long L. H., Halliwell B. (2011). Artefacts in cell culture: *α*-Ketoglutarate can scavenge hydrogen peroxide generated by ascorbate and epigallocatechin gallate in cell culture media. *Biochemical and Biophysical Research Communications*.

[15] Hou Y., Wang L., Ding B. (2011). Alpha-ketoglutarate and intestinal function. *Frontiers in Bioscience*.

[16] Mailloux R. J., Singh R., Brewer G., Auger C., Lemire J., Appanna V. D. (2009). *α*-ketoglutarate dehydrogenase and glutamate dehydrogenase work in tandem to modulate the antioxidant *α*-ketoglutarate during oxidative stress in Pseudomonas fluorescens. *Journal of Bacteriology*.

[17] Ali R., Mittal G., Sultana S., Bhatnagar A. (2012). Ameliorative potential of alpha-ketoglutaric acid (AKG) on acute lung injuries induced by ammonia inhalation in rats. *Experimental Lung Research*.

[18] Anthonymuthu T. S., Kenny E. M., Bayir H. (2016). Therapies targeting lipid peroxidation in traumatic brain injury. *Brain Research*.

[19] Velvizhi S., Nagalashmi T., Essa M. M., Dakshayani K. B., Subramanian P. (2002). Effects of *α*-ketoglutarate on lipid peroxidation and antioxidant status during chronic ethanol administration in wistar rats. *Polish Journal of Pharmacology*.

[20] Velvizhi S., Dakshayani K. B., Subramanian P. (2002). Effects of *α*-ketoglutarate on antioxidants and lipid peroxidation products in rats treated with ammonium acetate. *Nutrition Journal *.

[21] Dakshayani K. B., Subramanian P., Manivasagam T., Mohamed Essa M. (2006). Metabolic normalization of *α*-ketoglutarate against N-nitrosodiethylamine-induced hepatocarcinogenesis in rats. *Fundamental & Clinical Pharmacology*.

[22] Wang L., Xu Q., Wang C. (2017). Effects of dietary *α*-ketoglutarate supplementation on the antioxidant defense system and HSP 70 and HSP 90 gene expression of hybrid sturgeon Acipenser schrenckii ♀ × A. baerii ♂ exposed to ammonia-N stress. *Aquaculture Research*.

[23] KavasoğLu M., SarioğLu Y., Uysal K. (2015). Effect of sodium cyanide on antioxidant enzyme activities and lipid peroxidation in some tissues of mirror carp (Cyprinus carpio). *Pakistan Journal of Zoology*.

[24] Okafor P. N., Anyanwu V. O., Onyema H. O. (2010). The Effects of Cassava Cyanide on the Antioxidant (Glutathione) Status and Some Clinically Important Enzymes of Rats. *Journal of Pharmacology Toxicology*.

[25] Bhattacharya R., Satpute R. M., Hariharakrishnan J., Tripathi H., Saxena P. B. (2009). Acute toxicity of some synthetic cyanogens in rats and their response to oral treatment with alpha-ketoglutarate. *Food & Chemical Toxicology An International Journal Published for the British Industrial Biological Research Association*.

[26] Tulsawani R., Kumar D., Bhattacharya R. (2007). Effect of pre-treatment of *α*-ketoglutarate on cyanide-induced toxicity and alterations in various physiological variables in rodents. *Biomedical and Environmental Sciences*.

[27] Bhattacharya R., Rao P. V. L., Vijayaraghavan R. (2002). In vitro and in vivo attenuation of experimental cyanide poisoning by *α*-ketoglutarate. *Toxicology Letters*.

[28] Tulsawani R., Bhattacharya R. (2006). Effect of *α*-ketoglutarate on cyanide-induced biochemical alterations in rat brain and liver. *Biomedical and Environmental Sciences*.

[29] Hariharakrishnan J., Anand T., Satpute R. M., Jayaraj R., Prasad G. B. K. S., Bhattacharya R. (2009). Activity and gene expression profile of certain antioxidant enzymes in different organs of rats after subacute cyanide exposure: Effect of alpha-ketoglutarate. *Drug and Chemical Toxicology*.

[30] Bayliak M. M., Hrynkiv O. V., Knyhynytska R. V., Lushchak V. I. (2017). Alpha-ketoglutarate enhances freeze–thaw tolerance and prevents carbohydrate-induced cell death of the yeast Saccharomyces cerevisiae. *Archives of Microbiology*.

[31] Yin J., Wu M., Li Y. (2017). Toxicity assessment of hydrogen peroxide on Toll-like receptor system, apoptosis, and mitochondrial respiration in piglets and IPEC-J2 cells. *Oncotarget *.

[32] Guo S., Duan R., Wang L. (2017). Dietary *α*-ketoglutarate supplementation improves hepatic and intestinal energy status and anti-oxidative capacity of Cherry Valley ducks. *Animal Science Journal*.

[33] Jiang Q., Liu G., Wang X. (2017). Mitochondrial pathway is involved in the protective effects of alpha-ketoglutarate on hydrogen peroxide induced damage to intestinal cells. *Oncotarget *.

[34] Bayliak M. M., Shmihel H. V., Lylyk M. P. (2015). Alpha-ketoglutarate attenuates toxic effects of sodium nitroprusside and hydrogen peroxide in *Drosophila melanogaster*. *Environmental Toxicology and Pharmacology*.

[35] Radzki R. P., Bienko M., Pierzynowski S. G. (2012). Anti-osteopenic effect of alpha-ketoglutarate sodium salt in ovariectomized rats. *Journal of Bone and Mineral Metabolism*.

[36] Hou Y., Wang L., Ding B. (2010). Dietary *α*-ketoglutarate supplementation ameliorates intestinal injury in lipopolysaccharide-challenged piglets. *Amino Acids*.

[37] Mullen A., Hu Z., Shi X. (2014). Oxidation of alpha-ketoglutarate is required for reductive carboxylation in cancer cells with mitochondrial defects. *Cell Reports*.

[38] Zhang H., Ji S., Chen Y., Yang B., Xu X., Hu C. (2011). Effect of *α*-ketoglutaric acid on in vitro gas production, ruminal fermentation, and bacterial diversity. *Animal Feed Science & Technology*.

[39] Wu G., Bazer F. W., Davis T. A. (2009). Arginine metabolism and nutrition in growth, health and disease. *Amino Acids*.

[40] Xiao D., Zeng L., Yao K., Kong X., Wu G., Yin Y. (2016). The glutamine-alpha-ketoglutarate (AKG) metabolism and its nutritional implications. *Amino Acids*.

[41] Bayliak M. M., Shmihel H. V., Lylyk M. P., Storey K. B., Lushchak V. I. (2016). Alpha-ketoglutarate reduces ethanol toxicity in Drosophila melanogaster by enhancing alcohol dehydrogenase activity and antioxidant capacity. *Alcohol*.

[42] Bayliak M. M., Lylyk M. P., Shmihel H. V. (2016). Dietary alpha-ketoglutarate increases cold tolerance in *Drosophila melanogaster* and enhances protein pool and antioxidant defense in sex-specific manner. *Journal of Thermal Biology*.

[43] Wang L., Hou Y., Yi D. (2015). Dietary supplementation with glutamate precursor *α*-ketoglutarate attenuates lipopolysaccharide-induced liver injury in young pigs. *Amino Acids*.

[44] Mullen A., Hu Z., Shi X. (2014). Oxidation of alpha-ketoglutarate is required for reductive carboxylation in cancer cells with mitochondrial defects. *Cell Reports*.

[45] Niemiec T., Sikorska J., Harrison A. (2011). Alpha-ketoglutarate stabilizes redox homeostasis and improves arterial elasticity in aged mice. *Journal of Physiology and Pharmacology*.

[46] Whillier S., Garcia B., Chapman B. E., Kuchel P. W., Raftos J. E. (2011). Glutamine and *α*-ketoglutarate as glutamate sources for glutathione synthesis in human erythrocytes. *FEBS Journal*.

[47] Varma S. D., Hegde K. R. (2004). Effect of *α*-ketoglutarate against selenite cataract formation. *Experimental Eye Research*.

[48] Kovalenko T. N., Ushakova G. A., Osadchenko I., Skibo G. G., Pierzynowski S. G. (2011). The neuroprotective effect of 2-oxoglutarate in the experimental ischemia of hippocampus. *Journal of Physiology and Pharmacology An Official Journal of the Polish Physiological Society*.

[49] Chin R. M., Fu X., Pai M. Y. (2014). The metabolite alpha-ketoglutarate extends lifespan by inhibiting the ATP synthase and TOR. *Nature*.

[50] Bayliak M. M., Lylyk M. P., Shmihel H. V. (2017). Dietary alpha-ketoglutarate promotes higher protein and lower triacylglyceride levels and induces oxidative stress in larvae and young adults but not in middle-aged *Drosophila melanogaster*. *Comparative Biochemistry and Physiology—Part A : Molecular and Integrative Physiology*.

[51] Puntel R. L., Roos D. H., Grotto D., Garcia S. C., Nogueira C. W., Batista Teixeira Rocha J. (2007). Antioxidant properties of Krebs cycle intermediates against malonate pro-oxidant activity in vitro: A comparative study using the colorimetric method and HPLC analysis to determine malondialdehyde in rat brain homogenates. *Life Sciences*.

[52] Bayliak M., Burdyliuk N., Lushchak V. (2017). Growth on alpha-ketoglutarate increases oxidative stress resistance in the yeast* Saccharomyces cerevisiae*. *International Journal of Microbiology*.

[53] Cheng Z.-G., Zhang G.-D., Shi P.-Q., Du B.-S. (2013). Expression and antioxidation of Nrf2/ARE pathway in traumatic brain injury. *Asian Pacific Journal of Tropical Medicine*.

[54] Wang A. L., Niu Q., Shi N. (2015). Glutamine ameliorates intestinal ischemia-reperfusion Injury in rats by activating the Nrf2/Are signaling pathway. *International Journal of Clinical and Experimental Pathology*.

[55] Venoji R., Amirtharaj G. J., Kini A., Vanaparthi S., Venkatraman A., Ramachandran A. (2015). Enteral glutamine differentially regulates Nrf 2 along the villus-crypt axis of the intestine to enhance glutathione levels. *Journal of Gastroenterology and Hepatology*.

